# Statins to mitigate cardiotoxicity in cancer patients treated with anthracyclines and/or trastuzumab: a systematic review and meta-analysis

**DOI:** 10.1007/s10552-021-01487-1

**Published:** 2021-08-18

**Authors:** Mary Obasi, Arielle Abovich, Jacqueline B. Vo, Yawen Gao, Stefania I. Papatheodorou, Anju Nohria, Aarti Asnani, Ann H. Partridge

**Affiliations:** 1 Department of Medicine, David Geffen School of Medicine, University of California, Los Angeles, USA; 2 Department of Medicine, Johns Hopkins Hospital, Baltimore, MD USA; 3 Division of Cancer Epidemiology & Genetics, National Cancer Institute, Rockville, MD USA; 4 Analysis Group, Los Angeles, CA USA; 5 Harvard T.H. Chan School of Public Health, Boston, MA USA; 6 Dana-Farber/Brigham and Women’s Cancer Center, Boston, MA USA; 7 Harvard Medical School, Boston, MA USA; 8 CardioVascular Institute, Beth Israel Deaconess Medical Center and Harvard Medical School, Boston, MA USA; 9 Medical Oncology, Dana-Farber Cancer Institute, Boston, MA USA

**Keywords:** Statins, Cardiotoxicity, Cancer survivors, Meta-analysis

## Abstract

**Purpose:**

Cardiotoxicity affects 5–16% of cancer patients who receive anthracyclines and/or trastuzumab. Limited research has examined interventions to mitigate cardiotoxicity. We examined the role of statins in mitigating cardiotoxicity by performing a systematic review and meta-analysis of published studies.

**Methods:**

A literature search was conducted using PubMed, Embase, Web of Science, ClinicalTrials.gov, and Cochrane Central. A random-effect model was used to assess summary relative risks (RR), weighted mean differences (WMD), and corresponding 95% confidence intervals. Testing for heterogeneity between the studies was performed using Cochran’s *Q* test and the *I*^2^ test.

**Results:**

Two randomized controlled trials (RCTs) with a total of 117 patients and four observational cohort studies with a total of 813 patients contributed to the analysis. Pooled results indicate significant mitigation of cardiotoxicity after anthracycline and/or trastuzumab exposure among statin users in cohort studies [RR = 0.46, 95% CI (0.27–0.78), *p* = 0.004, ${ }I^{2}$ = 0.0%] and a non-significant decrease in cardiotoxicity risk among statin users in RCTs [RR = 0.49, 95% CI (0.17–1.45), *p* = 0.20, $I^{2}$ = 5.6%]. Those who used statins were also significantly more likely to maintain left ventricular ejection fraction compared to baseline after anthracycline and/or trastuzumab therapy in both cohort studies [weighted mean difference (WMD) = 6.14%, 95% CI (2.75–9.52), *p* < 0.001, $I^{2}$ = 74.7%] and RCTs [WMD = 6.25%, 95% CI (0.82–11.68, *p* = 0.024, $I^{2}$ = 80.9%]. We were unable to explore publication bias due to the small number of studies.

**Conclusion:**

This meta-analysis suggests that there is an association between statin use and decreased risk of cardiotoxicity after anthracycline and/or trastuzumab exposure. Larger well-conducted RCTs are needed to determine whether statins decrease risk of cardiotoxicity from anthracyclines and/or trastuzumab.

**Trial Registration Number and Date of Registration:**

PROSPERO: CRD42020140352 on 7/6/2020.

**Supplementary Information:**

The online version contains supplementary material available at 10.1007/s10552-021-01487-1.

## Introduction

Cardiotoxicity is the development of cardiovascular disease or damage to the heart resulting from cancer treatment. Anthracyclines and trastuzumab have demonstrated effectiveness in treating various cancers; however, both are associated with cardiotoxicity, resulting in an asymptomatic decline in left ventricular (LV) ejection fraction or subsequent development of heart failure. The estimated incidence of anthracycline and/or trastuzumab cardiotoxicity ranges from 5 to 16%, depending on various risk factors [1]. Several studies have examined the risk factors impacting this association among cancer patients [2, 3], yet few have examined prevention or mitigation strategies [4–8].

While the mechanisms of cardiotoxicity are not fully understood, it is hypothesized that anthracyclines cause oxidative damage to cardiac tissues resulting in cardiac cell death, which can ultimately lead to heart failure. If detected late, this cardiac damage can be irreversible [9]. Trastuzumab is primarily given to women with HER2-overexpressing breast cancer to block HER2 signaling, and in doing so it suppresses autophagy in cardiomyocytes to trigger accumulation of toxic reactive oxygen species (ROS) within cardiomyocytes [10]. Trastuzumab-induced LV dysfunction can often be reversible with discontinuation of therapy, and many patients can tolerate re-challenge after institution of neurohormonal antagonists for heart failure [10]. Often, anthracyclines and trastuzumab may be given sequentially, resulting in a significantly higher risk of cardiotoxicity [11].

Statins, also known as hydroxymethyl glutaryl coenzyme A reductase (HMG-CoA) inhibitors, are clinically well established for cardiovascular disease prevention due to their anti-inflammatory, anti-oxidative, and cholesterol-lowering effects [12]. Beyond these properties, statins have pleiotropic effects via inhibition of small Ras homologous (Rho) GTPases. These effects reduce topoisomerase II inhibition and the generation of reactive oxygen species, both mechanisms implicated in the pathophysiology of anthracycline and/or trastuzumab-induced cardiotoxicity. Therefore, statins may mitigate the risk of cardiotoxicity among cancer patients receiving these therapies [13].

The aim of this meta-analysis is to summarize the literature on statins as a mitigation modality for cardiotoxicity among cancer survivors treated with anthracyclines and/or trastuzumab and compile evidence on their effectiveness. The research question is: Does statin use lower the risk of cardiotoxicity among cancer survivors treated with anthracyclines and/or trastuzumab?

## Methods

This systematic review and meta-analysis were performed following the Preferred Reporting Items for Systematic Reviews and Meta-Analyses (PRISMA) guidelines [14, 15]. Ethical approval was not necessary because this study was a meta-analysis. The meta-analysis protocol was registered on PROSPERO: CRD42020140352 on July 7, 2020.

### Literature search strategy

A literature search was conducted in the databases Pubmed, Embase, Web of Science, ClinicalTrials.gov, and Cochrane Central using predefined keywords to identify articles examining statin use as a preventive method for the development of cardiotoxicity among cancer patients treated with anthracyclines and/or trastuzumab. See Supplementary Table 1 for the search criteria for each database. We used both medical subject headings and text words in PubMed, and we used Emtree terms and text words in Embase. We used text words in Web of Science, and ClinicalTrials.gov. Additionally, reference lists were reviewed for additional studies.

### Selection criteria

Two reviewers independently selected eligible studies (M. Obasi, A. Abovich). Disagreements between selected studies were settled after discussing with a third reviewer (J. Vo). The inclusion criteria consisted of studies which included cancer patients treated with anthracyclines and/or trastuzumab, and where statins were used during cancer treatment. The exclusion criteria consisted of case reports, reviews, guidelines, editorials, letters, and non-human research.

### Data extraction

Titles and/or abstracts were screened independently by two reviewers (M. Obasi, A. Abovich), based on the inclusion and exclusion criteria. The full texts of potentially eligible articles were screened independently by the same two authors for eligibility. The following information was obtained for this study-level meta-analysis: sample size, country of origin, details of the intervention and control conditions, study methodology, study settings, and outcomes. The reviewers (M. Obasi, A. Abovich) extracted and screened articles independently. Discrepancies were discussed to reach consensus.

### Variables

We identified studies that included patients taking any of the following statins: fluvastatin, atorvastatin, lovastatin, pravastatin, pitavastatin, rosuvastatin, and simvastatin. Study populations included patients who were diagnosed with any type of cancer and treated with either anthracyclines, trastuzumab, or both.

The primary outcome was the development of cardiotoxicity, defined as (1) incident heart failure diagnoses or (2) cardiotoxicity defined as reduction in LV ejection fraction of > 10% to < 55% without symptoms of heart failure, or > 5% drop to < 55% with symptoms, or reduction in LV ejection fraction to < 50%, by the Cardiac Review and Evaluation Committee [16]. The secondary outcome was mean change in LV ejection fraction from the pretreatment (baseline) value to follow-up.

### Methodological quality assessment

We assessed the quality of randomized controlled trials (RCTs) using version 2 of the Cochrane risk-of-bias tool for randomized trials (RoB 2). The following domains were critiqued: (1) bias arising from the randomization process, (2) bias due to deviations from intended interventions, (3) bias due to missing outcome data, (4) bias in measurement of the outcome, and (5) bias in selection of the reported result. Each was graded “low risk of bias (+)”, “high risk of bias (*x*),” or “some concerns (−).”

The Newcastle–Ottawa Scale has eight items that are categorized into three perspectives: selection of study groups (four items, one star each), comparability of the groups (one item, up to two stars), and ascertainment of the outcome (three items, one star each). A ‘‘star’’ represents a ‘‘high-quality’’ choice of individual study. Two reviewers independently selected eligible studies (M. Obasi, A. Abovich). Disagreements about selected studies were settled after further discussion.

### Data synthesis and analysis

We conducted two separate analyses examining the primary and secondary outcomes. First, we analyzed pooled relative risks using study-specific estimates for incidence of cardiotoxicity. Inverse variance weighting was used for the random-effects model.

For the secondary outcome, we examined the weighted mean difference (WMD) for change in LV ejection fraction using the random-effects model with inverse variance weighting. The WMD for LV ejection fraction was the difference in ejection fractions from baseline to follow-up compared between groups. Statistical significance was set at alpha = 0.05. All analyses were conducted with Stata version 15.

Testing for heterogeneity between the studies was performed using Cochran’s *Q* test [17] and the *I*^2^ test [18]. A *p* value of < 0.05 or an *I*^2^ higher than 50% were considered significant evidence of heterogeneity. Publication bias was not assessed due to the small number of studies.

## Results

### Search results and characteristics of included studies

**Fig. 1:**
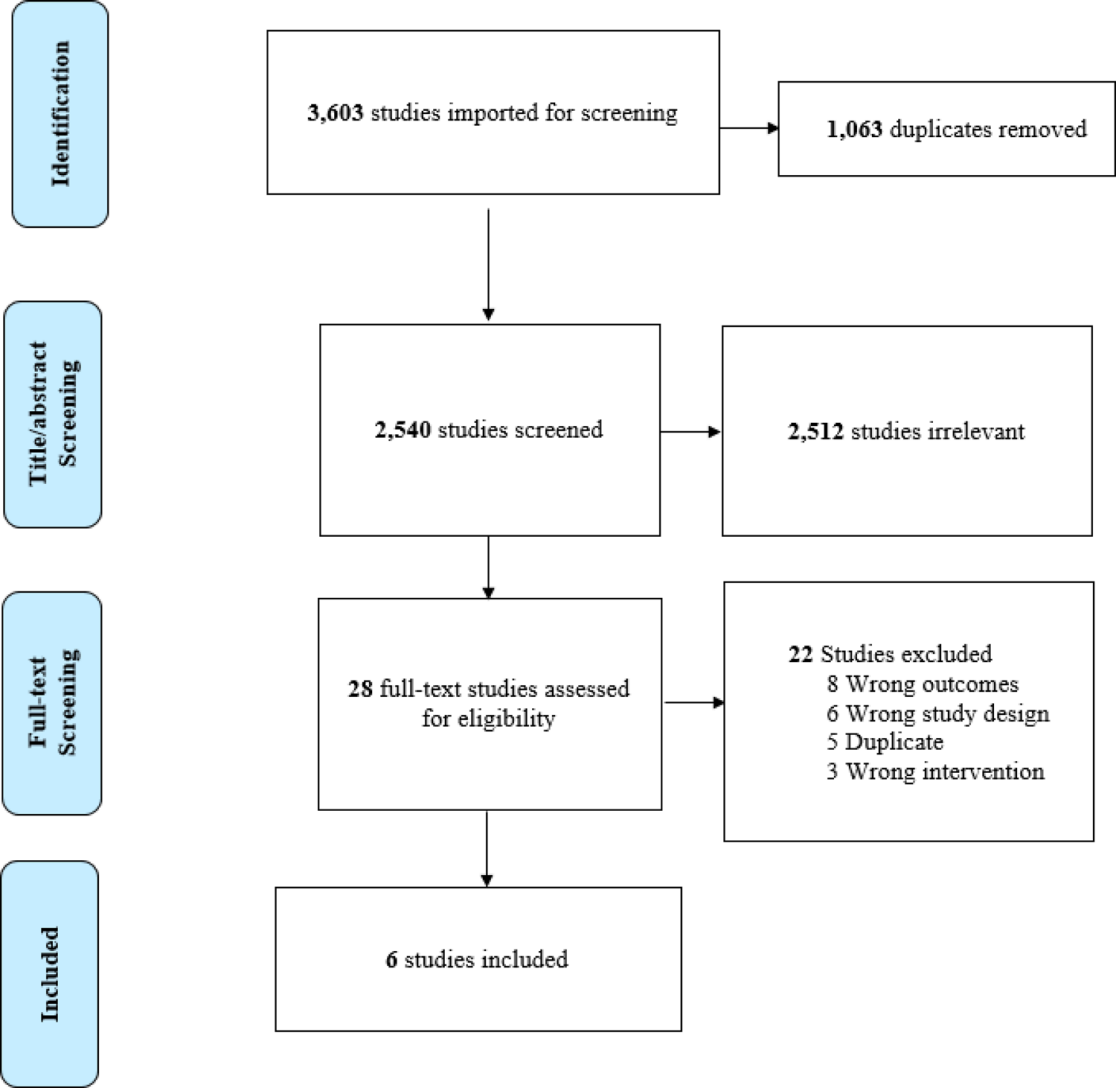
Flow diagram of screened, excluded, and analyzed publications

The initial database search yielded a total of 3,603 records, as shown in the PRISMA flow diagram (Fig. 1). There were 1,068 duplicates removed. After screening the titles and abstracts of the remaining studies (M. Obasi, A. Abovich), 28 records were identified. After more detailed evaluation, 22 records were excluded for details listed in Fig. 1. A total of six studies were included in the meta-analysis, including four observational cohort studies with a total of 813 patients [19–22] and two RCTs with a total of 117 patients [23, 24].

Table 1 describes the study characteristics. The age of participants in the RCTs ranged from 35 to 69 years. One RCT randomized newly diagnosed breast cancer patients to daily rosuvastatin or placebo starting the day before treatment with anthracycline and/or trastuzumab [23]. The other RCT randomized patients with various hematologic malignancies to daily atorvastatin or no statin prior to the initiation of anthracycline based therapy [24]. Both RCTs followed patients through 6 months of cancer treatment. Fifty percent (58/117) of patients in the two RCTs were randomized to statin therapy.

**Table 1:** Study characteristics

Author (year)	Study design	Statin group	No statin group	Study length	Mean age (years)	Male, *n* (%)	Statin dose, range (prevalence %)	Cancer	Cancer therapy	Matching criteria	Country
Calvillo-Arguelles (2019)	Obs	43	86	11 months (IQR 9, 18)	62.0 ± 9.0	0 (0)	Atorvastatin: 20 mg^a^ 10–40 (55.8%) Rosuvastatin: 10 mg^a^ 5–20 (25.6%) Simvastatin: 20 mg^a^ 10–40 (11.6%) Pravastatin: 20 mg^a^ 10–20 (7.0%)	Breast	Trastuzumab	Matched on age and anthracycline exposure status (1:2)	Canada
Chotenimitkhun (2015)	Obs	14	37	6 months	48.0 ± 2.0	18 (54.5)	Atorvastatin (35.7%) Simvastatin (64.3%) 40 ± 5 mg, 5–80	Breast Leukemia Lymphoma	Anthracycline	–	USA
Tase (2013)	Obs	144	288	2.55 years ± 1.68	53.5 ± 11.2	51 (11.8)	Rosuvastatin (53.5%) Atorvastatin (36.1%) Other statin (10.4%)	Breast Gastric	Anthracycline	Matched on propensity score (1:2)	Romania
Seicean (2012)	Obs	67	134	2.6 years ± 1.7	51.5 ± 10.8	0 (0)	NA	Breast	Anthracycline	Matched on propensity score (1:2)	USA
Nabati (2019)	RCT	38	39	6 months	49.2 ± 11.2	0 (0)	Rosuvastatin 20 mg	Breast	Anthracycline Trastuzumab	Randomized to statin or placebo on 1:1 ratio	Iran
Acar (2011)	RCT	20	20	6 months	53.0 ± 15.0	17 (42.5)	Atorvastatin 40 mg	Non-Hodgkin's lymphoma Multiple myeloma Leukemia	Anthracycline	Randomized to statin or no statin on 1:1 ratio	Turkey

*Obs* observational, *RCT* randomized control trial, *NA* not available

^a^Median dose

The age of participants in the observational cohort studies ranged from 40 to 70 years. These studies included patients with breast cancer, gastric cancer, leukemia, and lymphoma who had received treatment with anthracyclines and/or trastuzumab. Study duration ranged from 6 months to over 4 years. One-third (268/813) of participants in the cohort studies were exposed to statins during cancer therapy. One study specifically excluded patients with prior heart failure [22]. In all cohort studies, patients receiving statins had a higher prevalence of cardiovascular risk factors or established coronary artery disease [20, 22, 23]

The primary outcome, cardiotoxicity, was examined in three cohort studies [19, 20, 22] and two RCTs [23, 24]. Two cohort studies [19, 21] and two RCTs [23, 24] also examined the secondary outcome, mean change in LV ejection fraction. LV ejection fraction was measured by either echocardiography, multigated acquisition (MUGA) scan, or cardiovascular magnetic resonance imaging (cMRI) (Table 2).

**Table 2:** Cardiotoxicity definition and values, by study

Author (year)	Cardiac imaging	Statin group				Control group			
		Mean baseline LVEF (%)	Mean final LVEF (%)	Mean change in LVEF (%)	Cardiotoxicity incidence	Mean baseline LVEF (%)	Mean final LVEF (%)	Mean change in LVEF (%)	Cardiotoxicity incidence
Calvillo-Arguelles (2019)	MUGA	66.0 ± 7.0	64.6 (IQR 62.2, 67.1)	0 (IQR − 5, + 3)	5/43, 11.6%	66.7 ± 5.4	61.2 (IQR 59.6, 62.8)	− 6 (IQR − 10, − 1)	21/86, 24.4%
Nabati (2019)	Echocardiogram	55.05 ± 4.84	53.54 ± 6.68	− 1.5 ± 6.6	4/38, 10.5%	55.10 ± 5.09	49.95 ± 6.57	− 5.2 ± 5.7	6/39, 15.4%
Acar (2011)	Echocardiogram	61.3 ± 7.9	62.6 ± 9.3	+ 1.3 ± 3.8	1/20, 5%	62.9 ± 7.0	55.0 ± 9.5	− 7.9 ± 8.0	5/20, 25%
Chotenimitkhun (2015)	Cardiovascular MRI	56.6 ± 1.4	54.1 ± 1.3	+ 1.1 ± 2.6	–	57.5 ± 1.4	52.4 ± 1.2	− 6.5 ± 1.5	–
Tase (2013)	–	–	–	–	7/144, 4.9%	–	–	–	26/288, 9%
Seicean (2012)	–	–	–	–	4/67, 6%	–	–	–	23/134, 17.2%

*IQR* interquartile range, *MUGA* multigated acquisition scan, *MRI* magnetic resonance imaging, *LVEF* left ventricular ejection fraction

### Quality assessment results

**Fig. 2:**
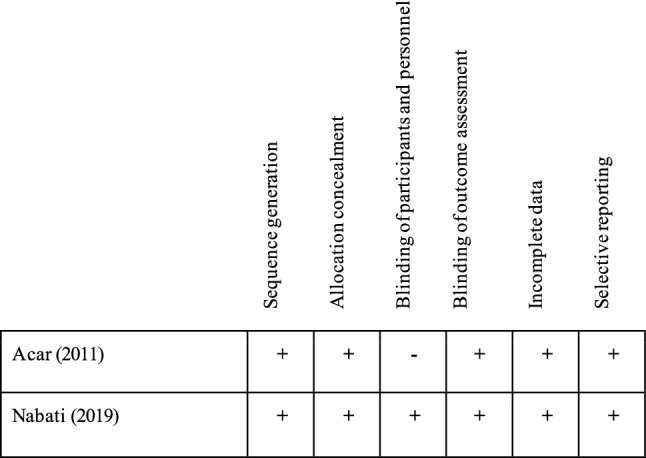
Methodological quality of randomized controlled trials: review authors’ opinion on each item of bias risk based on version 2 of the Cochrane risk-of-bias tool for randomized trials (RoB 2). Review authors’ opinion on each item of bias risk based on Cochrane handbook. ‘‘+ ’’, ‘‘*x*,’’ or ‘‘−’’ reflected low risk of bias, high risk of bias, and some concerns, respectively

Figure 2 shows our judgements from evaluating the five domains of the Cochrane risk-of-bias tool for the two RCTs. Most domains were at “low risk.” There was some concern about lack of blinding of participants and personnel in Acar et al. [24]. Table 3 summarizes the quality of the cohort studies based on Newcastle–Ottawa Scale scores. The scores ranged from 3 to 8, with a median of 7. Three studies were of high quality (≥ 6). The medians for each of the three perspectives were 2 for selection of study groups, 2 for comparability of the groups, and 3 for ascertainment of the outcome. Lower quality scores arose from the omission of information in Tase and colleagues since it was a conference abstract [20].

**Table 3:** Methodological quality of included cohort studies based on Newcastle–Ottawa Scale

Study	Selection	Comparability	Outcome	Total score
Calvillo-Arguelles (2019)	★★	★★	★★★	7
Chotenimitkhun (2015)	★★	★★	★★★	7
Tase (2013)		★	★★	3
Seicean (2012)	★★★	★★	★★★	8

### Incidence of cardiotoxicity

Five out of six studies in our analysis included cardiotoxicity as a primary or secondary outcome. Calvillo-Arguelles et al. specifically defined cardiotoxicity using the two-part CERC definition as described above [19]. Tase et al. and Seician et al. defined cardiotoxicity as new onset heart failure hospitalization [20, 22]. Nabati et al. and Acar et al. defined cardiotoxicity as reduction in LVEF to < 45% and < 50%, respectively [23, 24].

Incidence of cardiotoxicity ranged from 5 to 12% among statin users, and 9–25% among the non-exposed group. Among cohort studies, statins significantly mitigated the risk of cardiotoxicity after anthracycline and/or trastuzumab exposure compared to no statin use [RR = 0.46, 95% CI (0.27–0.78), *p* = 0.004]. When examining the RCTs, we found a similarly lower relative risk of cardiotoxicity among statin users compared to no statin use, although these results did not achieve statistical significance [RR = 0.49, 95% CI (0.17–1.45), *p* = 0.200] (Table 4). There was no significant heterogeneity found between studies assessing incidence of cardiotoxicity, among both observational studies (*I*^2^ = 0.0%; *p* = 0.800) and RCTs (*I*^2^ = 5.6%; *p* = 0.300). Due to the small number of studies, publication bias was not assessed.

**Table 4:**
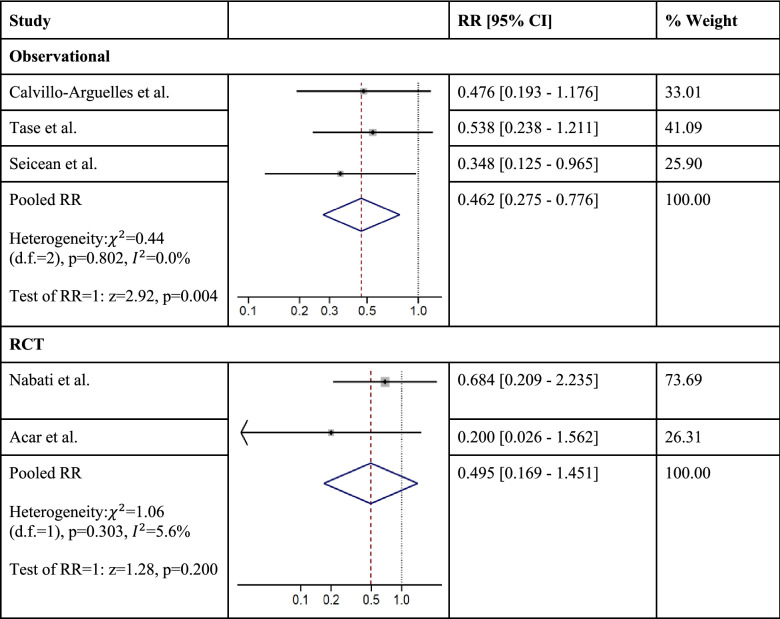
Pooled relative risk for incidence of cardiotoxicity using random-effects model

## Change in LV ejection fraction

Among statin users, the change in LV ejection fraction ranged from − 1.5 to + 1.3%. The range of change in LV ejection fraction was − 5.2 to − 7.9% among non-statin users. Those who used statins were also more likely to maintain LV ejection fraction at the same or higher percentages from baseline after anthracycline and/or trastuzumab exposure, compared to those who did not use statins in the cohort studies [WMD = 6.14%, 95% CI (2.75–9.52), *p* < 0.001] and RCTs [WMD = 6.25%, 95% CI (0.82–11.68), *p* = 0.024] (Table 5). There was significant heterogeneity found between studies assessing mean LVEF change, among both observational studies (*I*^2^ = 74.7%; *p* = 0.047) and RCTs (*I*^2^ = 80.9%; *p* = 0.022). We were not able to examine publication bias because we had less than 10 studies in our analysis.

**Table 5:**
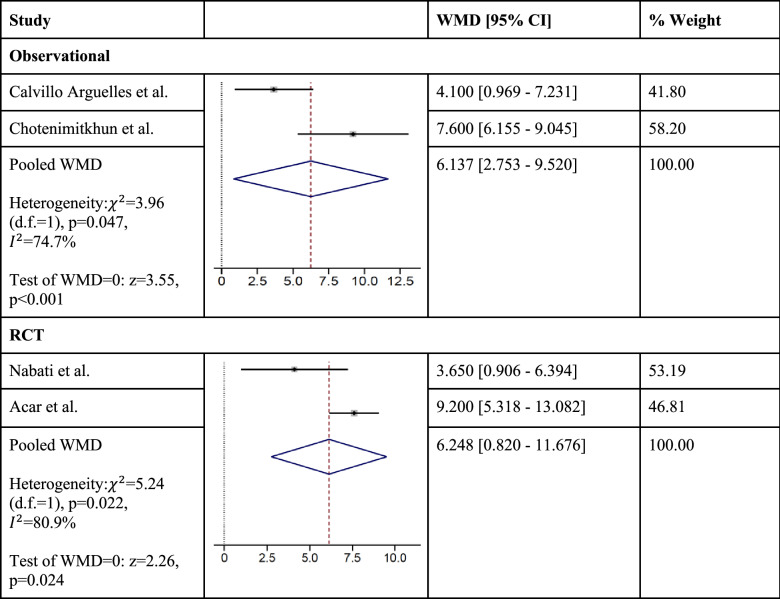
Estimated weighted mean difference for change in LV ejection fraction using random-effects model with inverse variance weights

## Discussion

In this study, we found that statin use was associated with a lower risk of cardiotoxicity among patients undergoing anthracycline and/or trastuzumab therapy. This meta-analysis demonstrated that the risk of cardiotoxicity was 50% lower among statin users, although the risk reduction was not significant in the included RCTs. Further, the analyses from the cohort and RCTs demonstrated a ~ 6% difference in the change in LV ejection fraction among statin users compared to those who did not receive statins, suggesting that cancer patients using statins were more likely to preserve their LV ejection fraction during anthracycline and/or trastuzumab therapy. Due to significant heterogeneity, the clinical implications of this are not clear. These findings were consistent across both cohort studies and RCTs.

Cardiotoxicity has many manifestations including subtle changes in myocardial strain or biomarkers, asymptomatic left ventricular systolic dysfunction, or clinical heart failure [25]. Our findings are consistent with prior meta-analyses that have included statins as one of various cardioprotective agents associated with maintaining LV ejection fraction and lowering the risk of cardiotoxicity among patients treated with anthracyclines and/or trastuzumab, compared to placebo or controls [26, 27]. Our study adds to this literature by specifically examining the potential therapeutic benefit of statin use among cancer patients treated with cardiotoxic chemotherapy. While incident cardiotoxicity was not significant in RCTs, our results suggest that change in LV ejection fraction can be mitigated by statin use even among patients who do not meet criteria for cardiotoxicity as currently defined. This highlights the importance of assessing both incident cardiotoxicity and LV ejection fraction changes.

Increasing awareness of cardiotoxicity among cancer survivors has led to the need for cardioprotective measures. Dexrazoxane is the only drug approved by the Federal Drug Administration to prevent chemotherapy-induced cardiotoxicity; however, concerns related to decreased cancer-specific survival and risk for secondary malignancy have limited its utilization in clinical practice [13, 28, 29]. In contrast, statins have demonstrated potential to reduce the risk of cardiotoxicity and may also be associated with reductions in cancer mortality [30].

Other cardioprotective agents, including beta-blockers (BB), angiotensin-converting enzyme (ACE) inhibitors, and angiotensin receptor blockers (ARBs), have demonstrated effectiveness in reducing the risk of cardiotoxicity among cancer patients, as evidenced by various RCTs. ACE inhibitors and BB were associated with increased cardiotoxicity-free survival and fewer interruptions in cancer therapy compared to placebo among HER2 positive breast cancer patients treated with trastuzumab (± anthracyclines) [4, 8]. Similarly, among patients treated for hematological cancers, concurrent use of enalapril and carvedilol with anthracyclines in the Prevention of Left Ventricular Dysfunction with Enalapril and Carvedilol in Patients Submitted to Intensive Chemotherapy for the Treatment of Malignant Hemopathies (OVERCOME) trial was associated with a lower incidence of cardiotoxicity and no significant change in LV ejection fraction compared to controls [7]. In contrast, in HER2 negative breast cancer patients treated with anthracyclines, carvedilol did not have an impact on cardiotoxicity incidence or LV ejection fraction [6]. Furthermore, in a trial of early stage breast cancer survivors who received anthracyclines, ARBs (candesartan) were associated with protection against LV dysfunction, whereas the BB (metoprolol) did not have an effect [5]. While there is a signal for reduced cardiotoxicity when using these cardioprotective agents, there is significant heterogeneity in the results making them inconclusive [31].

Patients receiving chemotherapy are especially at risk for hypotension from fluid shifts, infection, etc., and thus anti-hypertensive therapy with ACE inhibitors, ARBs, or BB may not be tolerated. While carvedilol has been shown by many studies to have an impact on reducing cardiotoxicity through its antioxidant and anti-apoptotic properties [32–38], it has also been shown to have side effects of symptomatic hypotension and dizziness [6]. Thus, statins may be a better tolerated and may be an appropriate adjunct or alternative to ACE inhibitors, ARBs, or BB for mitigating cardiotoxicity in patients receiving anthracyclines and/or trastuzumab.

Implications from this analysis include the need for large-scale RCTs to understand the impact of statins on preventing cardiotoxicity. Currently, there are several ongoing clinical trials that will hopefully provide clarity about the cardioprotective potential of statin therapy [39–41]. Additionally, there is a need to understand if and how statins may impact cancer treatment, although current research suggests that they are safe and may reduce recurrence and improve survival for certain malignancies such as breast cancer [42, 43]. Current clinical guidelines highlight the need for preventing and monitoring cardiotoxicity among cancer survivors, through suggested serial screening during treatment and up to 1–2 years post treatment [44–46]. Guidelines currently do not recommend routine concomitant use of cardioprotective drugs (i.e., statins, ACE inhibitors, ARBs, BB) with potentially cardiotoxic cancer therapies. Further data to support the use of statins, or other cardioprotective agents, in primary and/or secondary prevention of cardiotoxicity during treatment could have significant implications for the long-term outcomes of cancer patients.

To our knowledge, this study is the first meta-analysis to exclusively examine the impact of statins on mitigation of cardiotoxicity. We provide a pooled analysis of the available data examining statins as cardioprotective agents in cancer patients treated with anthracyclines and/or trastuzumab. However, this study has several limitations.

Due to the paucity of research examining statins as cardioprotective agents, the sample size of this meta-analysis is small. We attempted to overcome this limitation by including both RCTs and observational studies but it is important to note the potential for confounding among both RCTs and observational studies. The sample size of the RCTs included in our analysis were small; therefore, the effectiveness of randomization cannot be guaranteed. Though the RCTs attempted to address this issue by accounting for age, sex, cumulative doses of chemotherapy, and comorbidities that affect likelihood of statin use and/or cardiovascular risk, there are still possible confounders that were not accounted for, such as race and smoking status [23, 24, 44]. Though the observational studies included in our analysis also controlled for a robust number of confounders that are known to affect likelihood of statin exposure and/or LVEF through multivariable risk adjustment (e.g., age, body measurements, comorbidities, chemotherapy doses, and smoking status), there is still a possibility that there were sources of confounding that were not included in the models, such as race [19, 21, 22, 44]. We could not adjust for additional confounders in our meta-analysis because we did not have access to this data. There is also potential for selection bias with observational studies and RCTs. Among the RCTs, the risk of biased allocation to statin groups is unclear because information on the random allocation process is omitted. Without this information, there is a potential that those studies were not properly randomized and/or concealed which could have potentially led to recruitment based on favorable prognostic factors such as medication adherence and medical comorbidities. This limitation was overcome in Nabati et al., by providing distributions of baseline characteristics. Additionally, subjects involved in RCTs of preventative interventions tend to exclude subjects with worse prognosis and include more affluent, educated, and healthier participants [47]. Regarding observational studies, selection bias may occur if patients who did not use statins were healthier at baseline. Cancer patients who were already taking statins may have received prediagnostic (pre-cancer diagnosis) cardiovascular benefit [48]. As such, there is insufficient data to determine causation between statin use and lower risk of cardiotoxicity, warranting future larger RCTs randomizing by initiation of statin use to understand its clinical benefit. In addition, cardiotoxicity can develop many years after completion of chemotherapy. The RCTs may have underestimated the cardioprotective benefit of statin therapy due to their limited study duration (6 months). The inclusion of observational studies potentially strengthened our conclusions by allowing longer follow-up. Lastly, because of the small number of studies, we were not able to assess publication bias or conduct a meta-regression or other exploration of heterogeneity. The generalizability of these study findings should be considered in light of these limitations.

In conclusion, statins have the potential to mitigate the cardiotoxic effects of anthracyclines and trastuzumab. Based on these pooled results, cancer survivors on statin therapy during cancer treatment had a lower incidence of cardiotoxicity and were more likely to maintain LV ejection fraction during study follow-up. These data provide support for larger RCTs examining the safety and efficacy of statins as cardioprotective agents in patients receiving anthracycline and/or trastuzumab therapy.

## Supplementary Information

Below is the link to the electronic supplementary material.

Supplementary file1 (PDF 61 kb)
