# Supplementary material for: Statins to mitigate cardiotoxicity in cancer patients treated with anthracyclines and/or trastuzumab: a systematic review and meta-analysis
Source: Cancer Causes Control. 2021 Aug 18;32(12):1395–405. doi: 10.1007/s10552-021-01487-1 (PMC8541988; doi:10.1007/s10552-021-01487-1)
Supplement: Supplementary file 1 — Supplementary file1 (PDF 61 kb) [file 10552_2021_1487_MOESM1_ESM.pdf]

Table 1S.

List of search criteria

**Database**	**Search Terms**
Pubmed	("neoplasms"[mesh] OR cancer*[tiab] OR carcinoma*[tiab] OR neoplasm*[tiab] OR tumor[tiab] OR tumour[tiab] OR tumors[tiab] OR tumours[tiab] OR malignan*[tiab]) AND ("Antineoplastic Agents"[Mesh] OR "Antineoplastic Agents"[Pharmacological Action] OR chemotherap*[tiab] OR antineoplastic agent*[tiab] OR antineoplastic drug*[tiab] OR anticancer agent*[tiab] OR anticancer drug*[tiab] OR "Anthracyclines"[Mesh] OR daunorubicin[tiab] OR doxorubicin[tiab] OR epirubicin[tiab] OR Aclarubicin[tiab] OR carubicin[tiab] OR idarubicin[tiab] OR nogalamycin[tiab] OR plicamycin[tiab] OR "Trastuzumab"[Mesh] OR Trastuzumab[tiab] OR herceptin[tiab]) AND ("Hydroxymethylglutaryl-CoA Reductase Inhibitors"[Pharmacological Action] OR "Hydroxymethylglutaryl-CoA Reductase Inhibitors"[Mesh] OR "Fluvastatin"[mesh] OR statin*[tiab] OR hmg coa reductase[tiab] OR hydroxymethylglutaryl-coa reductase[tiab] OR atorvastatin[tiab] OR lovastatin[tiab] OR pravastatin[tiab] OR pitavastatin[tiab] OR rosuvastatin[tiab] OR fluvastatin[tiab] OR simvastatin[tiab])
Embase	('cancer':ab,ti OR carcinoma*:ab,ti OR neoplasm*:ab,ti OR tumor*:ab,ti OR tumour*:ab,ti OR malignan*:ab,ti) AND ('antineoplastic agent'/exp OR chemotherap*:ab,ti OR 'antineoplastic agent*':ab,ti OR (((antineoplastic OR anticancer) NEAR/3 (agent* OR drug*)):ab,ti) OR daunorubicin:ab,ti OR doxorubicin:ab,ti OR epirubicin:ab,ti OR plicamycin:ab,ti OR nogalamycin:ab,ti OR idarubicin:ab,ti OR carubicin:ab,ti OR aclarubicin:ab,ti OR trastuzumab:ab,ti OR herceptin:ab,ti) AND ((('hydroxymethylglutaryl coenzyme a reductase inhibitor'/exp OR statin*:ab,ti OR hmg) AND coa AND reductase:ab,ti OR 'hydroxymethylglutaryl coa') AND reductase:ab,ti OR atorvastatin:ab,ti OR lovastatin:ab,ti OR pravastatin:ab,ti OR pitavastatin:ab,ti OR rosuvastatin:ab,ti OR fluvastatin:ab,ti OR simvastatin:ab,ti)
Cochrane	“Statin” AND “breast cancer”
Web of Science	TS= (cancer* OR carcinoma* OR neoplasm* OR tumor OR tumour OR tumors OR tumours OR malignan*)<br>AND<br>TS=("Antineoplastic Agents" OR "Antineoplastic Agents" OR chemotherap* OR antineoplastic agent* OR antineoplastic drug* OR anticancer agent* OR anticancer drug* OR "Anthracyclines" OR daunorubicin OR doxorubicin OR epirubicin OR "Trastuzumab" OR Trastuzumab OR herceptin OR anthracyclines OR aclarubicin OR daunorubicin OR Carubicin OR Doxorubicin OR Idarubicin OR Nogalamycin OR Plicamycin)<br>AND<br>TS=("Hydroxymethylglutaryl-CoA Reductase Inhibitors" OR "Hydroxymethylglutaryl-CoA Reductase Inhibitors" OR "Fluvastatin" OR statin* OR hmg coa reductase OR hydroxymethylglutaryl-coa reductas OR atorvastatin OR lovastatin OR pravastatin OR pitavastatin OR rosuvastatin OR fluvastatin OR simvastatin)
ClinicalTrials.gov	Statin | Breast Cancer<br>(also searched for: Hydroxymethylglutaryl CoA Reductase Inhibitors, Breast Neoplasms, Neoplasm and more.)
